# Supplementary material for: Achieving large thermal hysteresis in an anthracene-based manganese(II) complex via photo-induced electron transfer
Source: Nat Commun. 2022 May 12;13:2646. doi: 10.1038/s41467-022-30425-1 (PMC9098415; doi:10.1038/s41467-022-30425-1)
Supplement: Supplementary file 1 — Supplementary Information [file 41467_2022_30425_MOESM1_ESM.pdf]

Supplementary Information for

Achieving Large Thermal Hysteresis of an Anthracene-Based Manganese(II) Complex via Photo-Induced Electron Transfer

Ji-Xiang Hu[1], Qi Li[1], Hai-Lang Zhu[2], Zhen-Ni Gao[1], Qian Zhang[1], Tao Liu[2]* and Guo-Ming Wang[1]*

[1]College of Chemistry and Chemical Engineering, Qingdao University, Shandong, 266071, P. R. China;

[2]State Key Laboratory of Fine Chemicals, Dalian University of Technology, Dalian, 116024, P. R. China.

*Corresponding Authors: Guo-Ming Wang: gmwang_pub@163.com; Tao Liu: liutao@dlut.edu.cn.

Supplementary Figures

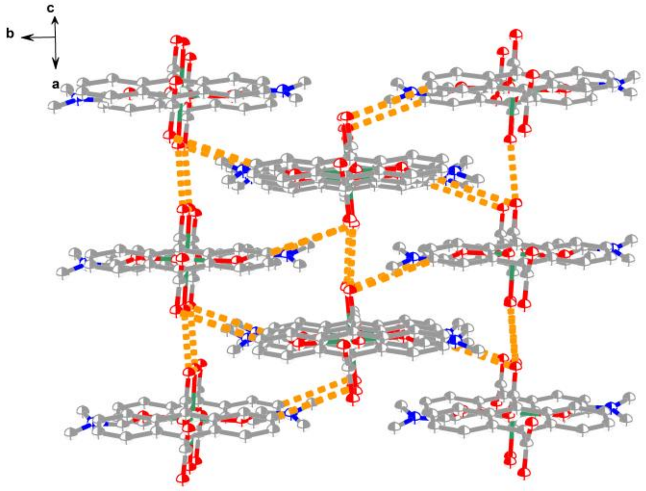

Supplementary Figure 1. The supramolecular structure via H-bond interactions for compound **1** with thermal ellipsoids set at 50% probability.

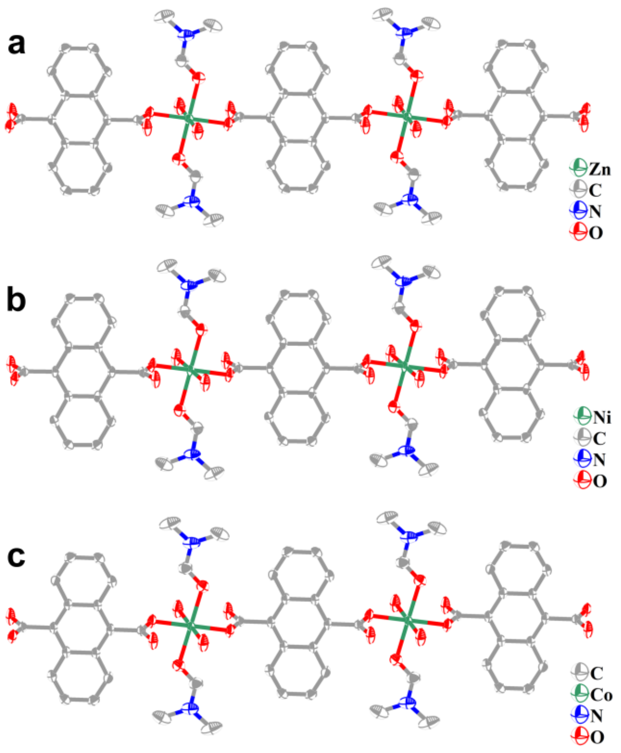

Supplementary Figure 2. The 1D structure for compounds **2** (**a**), **3** (**b**) and **4** (**c**) with thermal ellipsoids set at 50% probability. The green, grey-40%, red and blue colors represented M^{2+} (M = Zn, Ni and Co for **2**, **3** and **4**, respectively), C, O and N atoms, respectively. Hydrogen atoms are omitted for clarity.

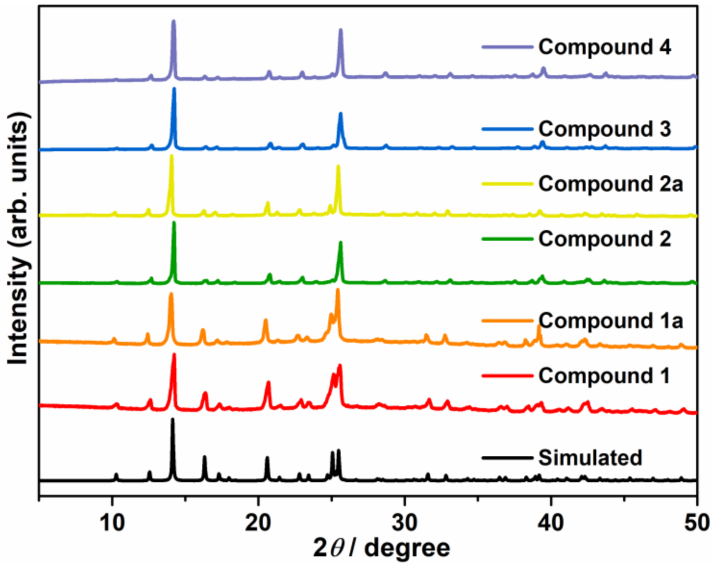

Supplementary Figure 3. PXRD plots for compounds **1**–**4** at 293 K.

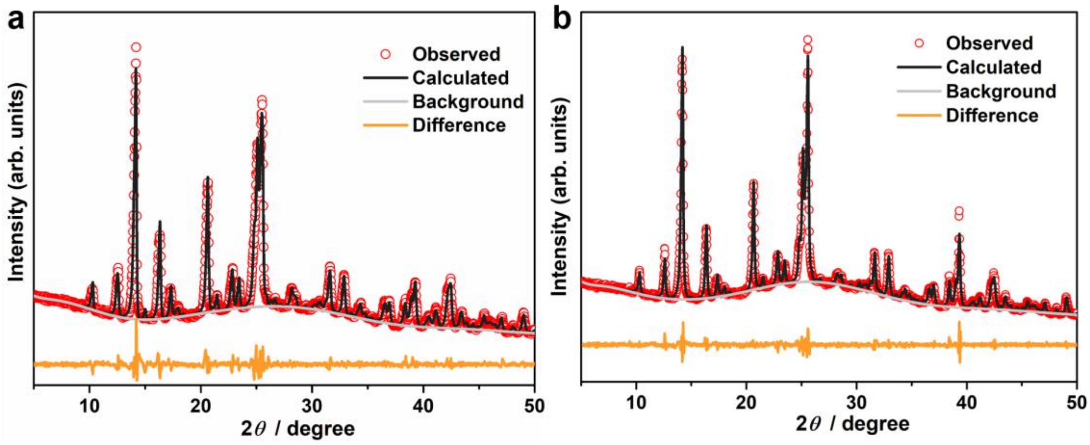

Supplementary Figure 4. Le Bail method fit to PXRD date for compounds **1** (**a**) and **1a** (**b**).

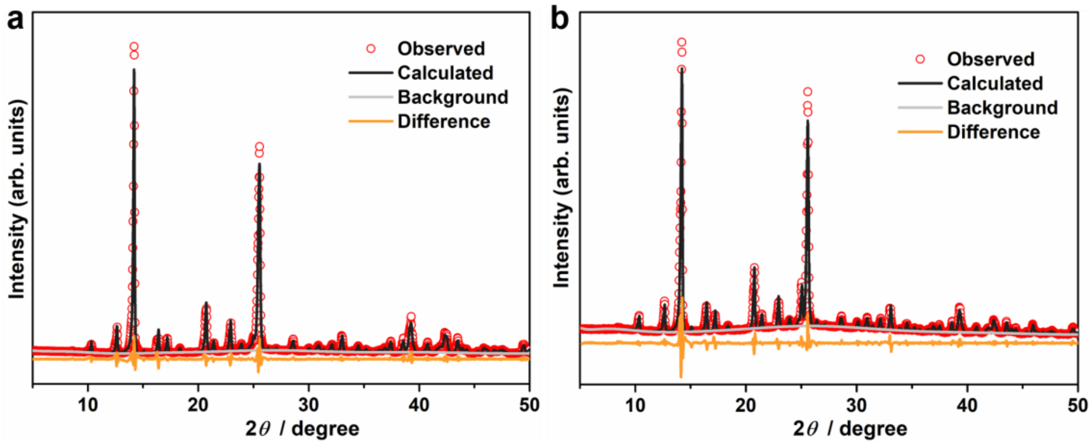

Supplementary Figure 5. Le Bail method fit to PXRD date for compounds **2** (**a**) and **2a** (**b**).

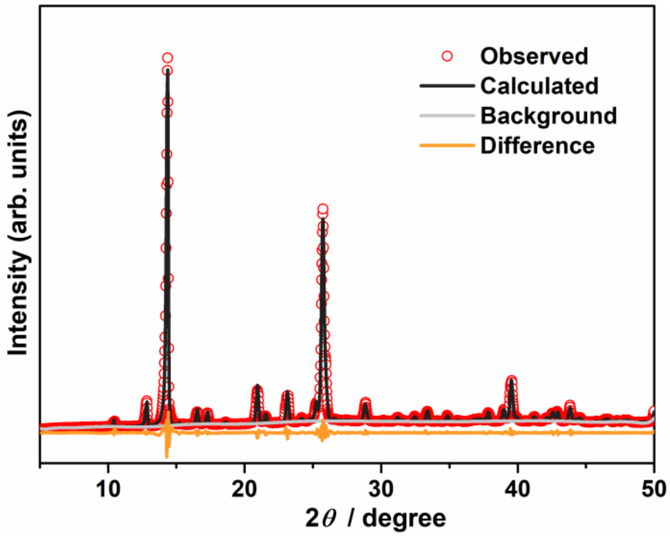

Supplementary Figure 6. Le Bail method fit to PXRD date for compound **3**.

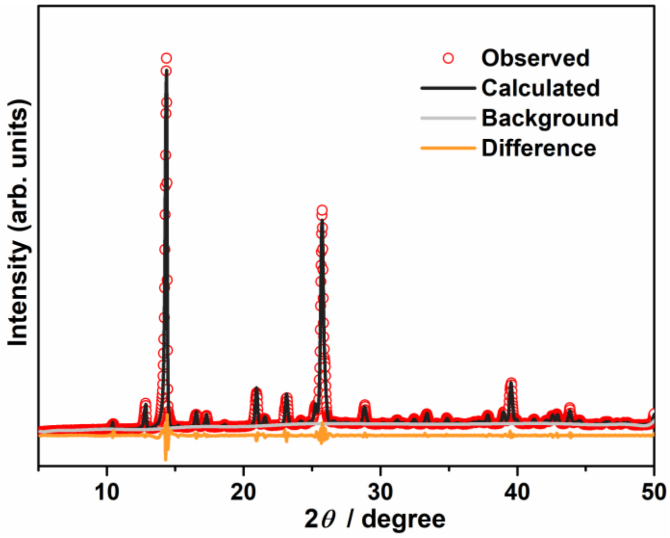

Supplementary Figure 7. Le Bail method fit to PXRD date for compound **4**.

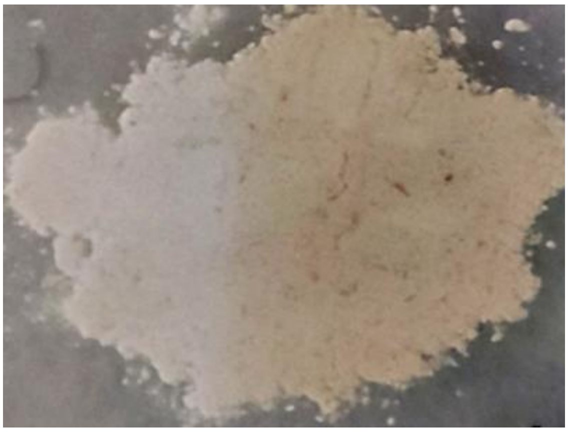

Supplementary Figure 8. The color changes of powder sample **1** after Xe lamp light irradiation.

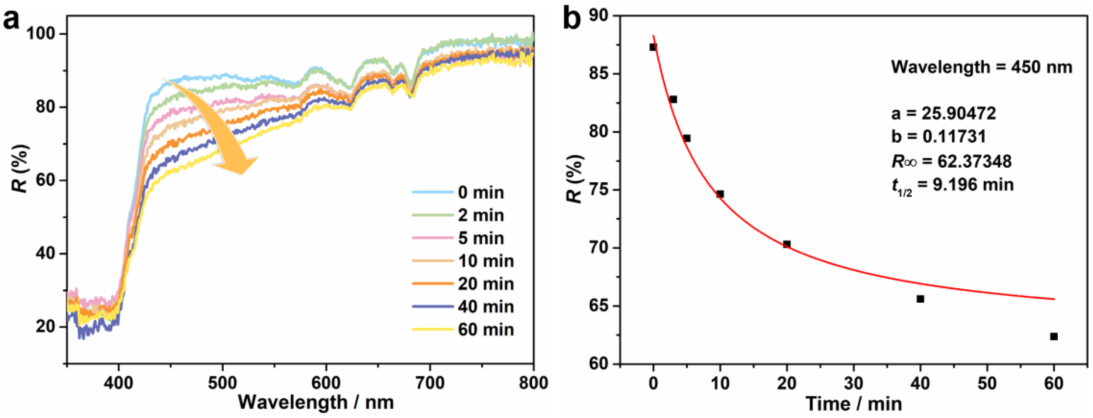

Supplementary Figure 9. (**a**) Time-dependent UV-Vis diffuse-reflectance spectra of **1** upon irradiation, which were obtained by transforming the absorption spectra; (**b**) The plot of relative intensity of time-dependent UV-Vis spectra at 450 nm upon light irradiation. The red solid line presented the fitted curve. The kinetics of the coloration for **1** was analyzed in order to estimate the rate of photochromism, using function $R^{\lambda\max}(t) = a/(bt + 1) + R^{\lambda\max}(\infty)$ to fit peak of diffuse reflection versus time curve. The relative parameters are listed in Supplementary Figure 9b, wherein, the constant a and b are proportionality constant, $R^{\lambda\max}(\infty)$ is the peak value of diffuse reflection spectrum after complete irradiation. Furthermore, substituting $R(t) = [R(0) + R(\infty)]/2$ into the function gives the value of half-time $t_{1/2}$ about 9.20 min.

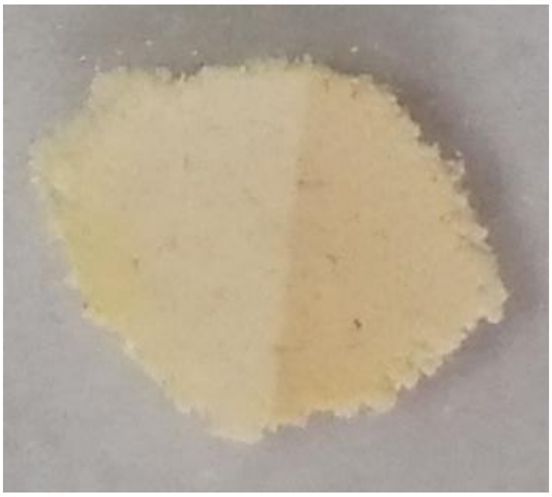

Supplementary Figure 10. The color changes of powder sample **2** after Xe lamp light irradiation.

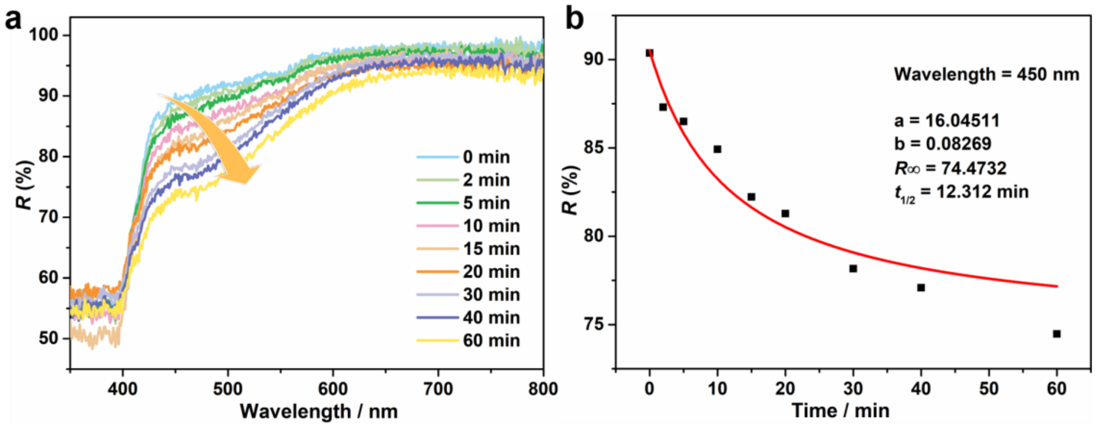

Supplementary Figure 11. (**a**) Time-dependent UV-Vis diffuse-reflectance spectra of **2** upon irradiation, which were obtained by transforming the absorption spectra; (**b**) The plot of relative intensity of time-dependent UV-Vis spectra at 450 nm upon light irradiation. The red solid line presented the fitted curve. The kinetics of the coloration for **2** was analyzed in order to estimate the rate of photochromism, using function $R^{\lambda\max}(t)$ = a/(bt + 1) + $R^{\lambda\max}(\infty)$ to fit peak of diffuse reflection versus time curve. The relative parameters are listed in supplementary Figure 11b, wherein, the constant a and b are proportionality constant, $R^{\lambda\max}(\infty)$ is the peak value of diffuse reflection spectrum after complete irradiation. Furthermore, substituting $R(t) = [R(0) + R(\infty)]/2$ into the function gives the value of half-time $t_{1/2}$ about 12.31 min.

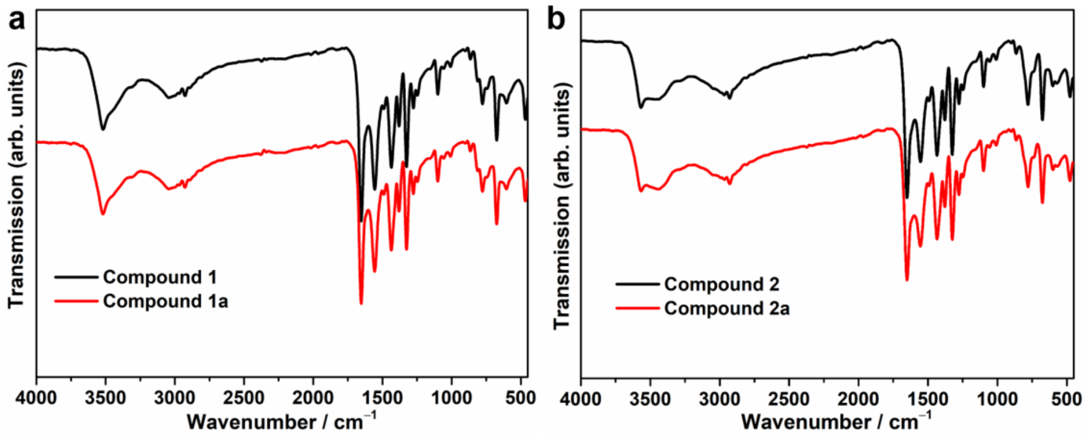

Supplementary Figure 12. IR plots for compounds **1** (**a**) and **2** (**b**).

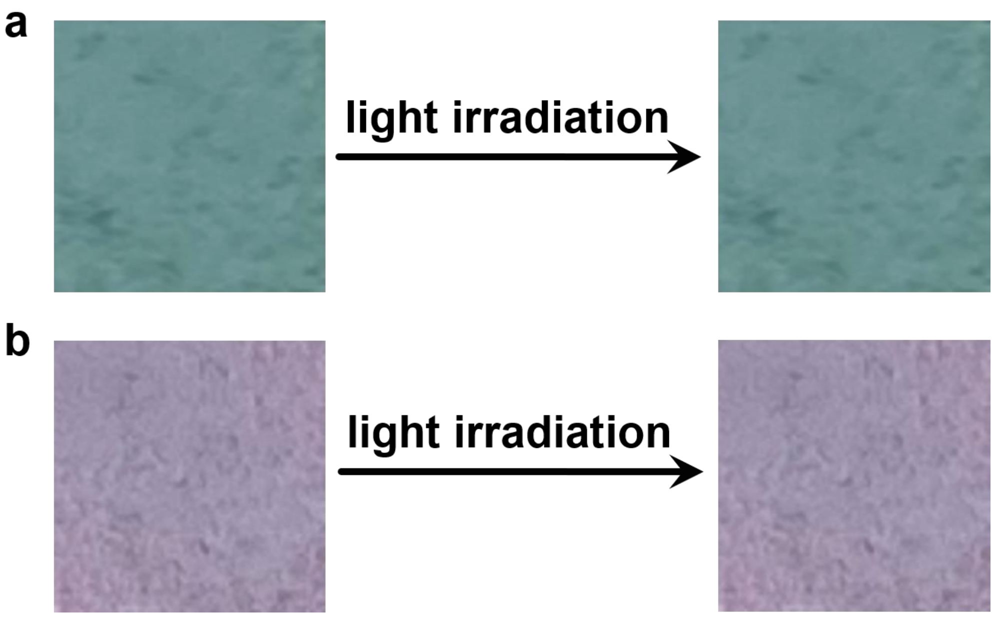

Supplementary Figure 13. The color of compounds **3** (**a**) and **4** (**b**) before and after Xe lamp light irradiation. As seen in the pictures, no observable photochromic phenomenon occurred in these two compounds.

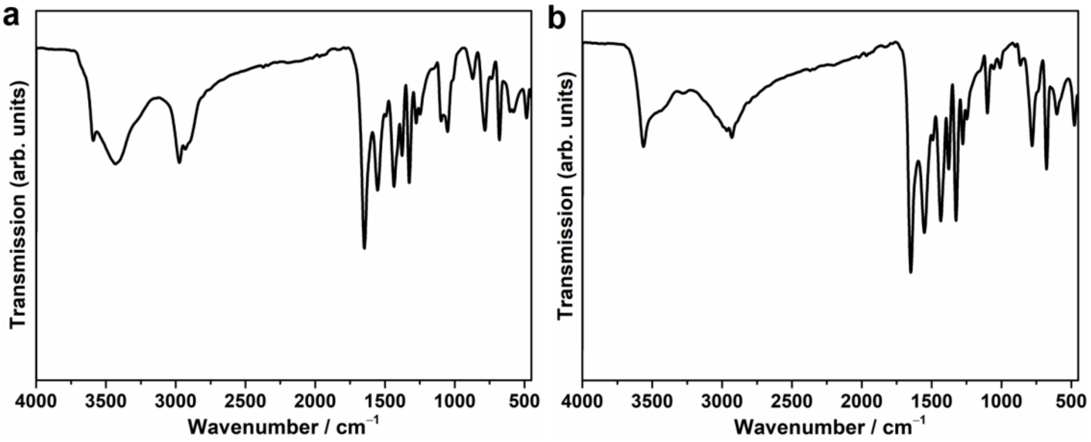

Supplementary Figure 14. IR plots for compounds **3** (**a**) and **4** (**b**) at 293 K.

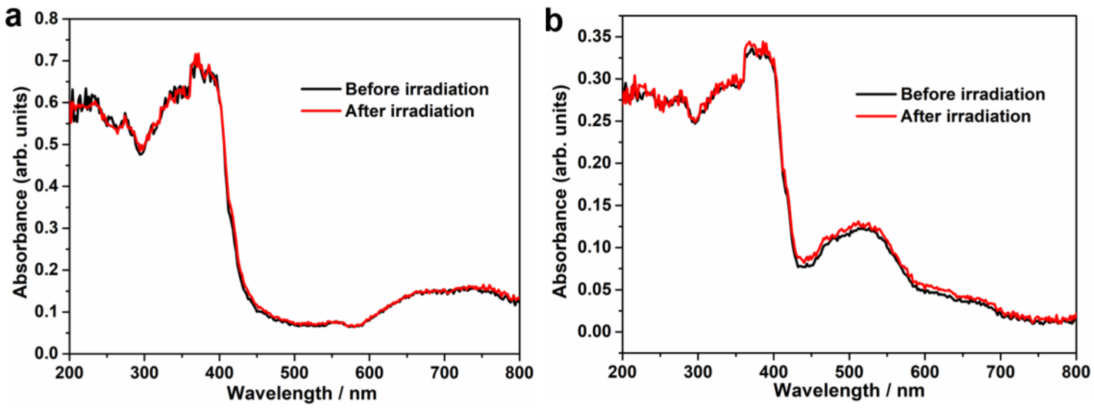

Supplementary Figure 15. Solid state UV-Vis spectra of compounds **3** (**a**) and **4** (**b**) before and after light irradiation. As seen in the spectra, no observable photochromic phenomenon occurred in these two compounds.

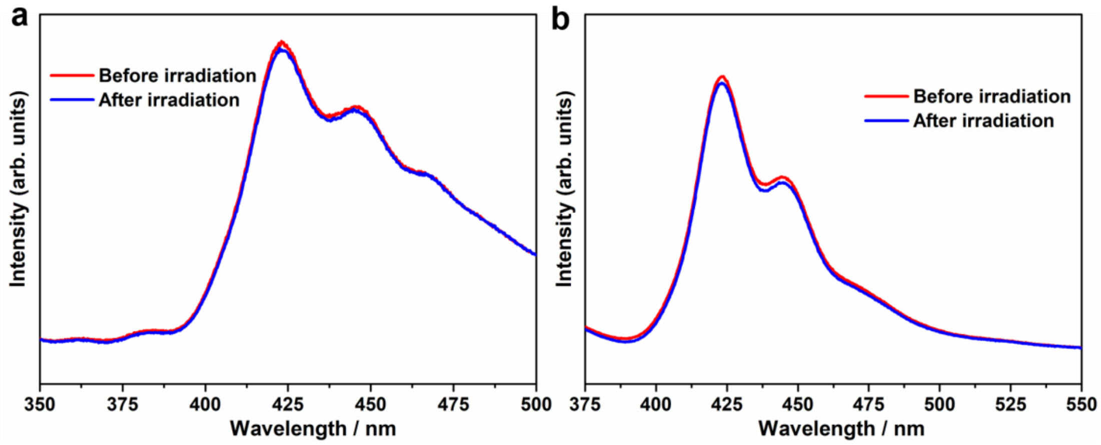

Supplementary Figure 16. Photoluminescent spectra of compounds **3** (**a**) and **4** (**b**) before and after light irradiation. As seen in the spectra, no observable photochromic phenomenon occurred in these two compounds.

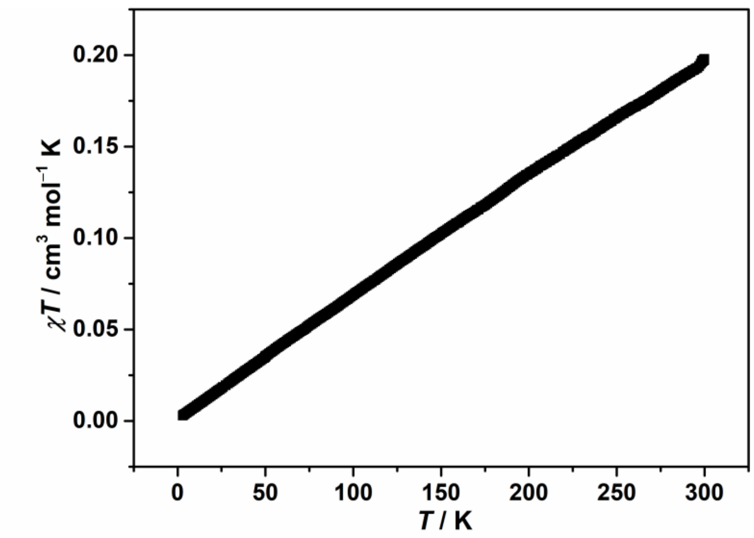

Supplementary Figure 17. Temperature-dependent susceptibilities of **2a** under a direct-current magnetic field of 1000 Oe.

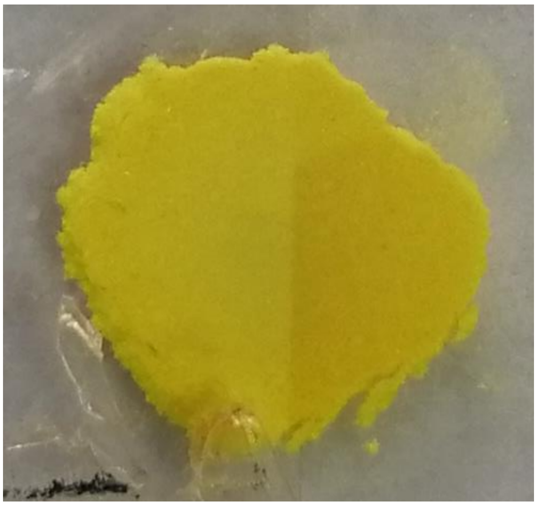

Supplementary Figure 18. The color changes of H_2ADC after Xe lamp light irradiation.

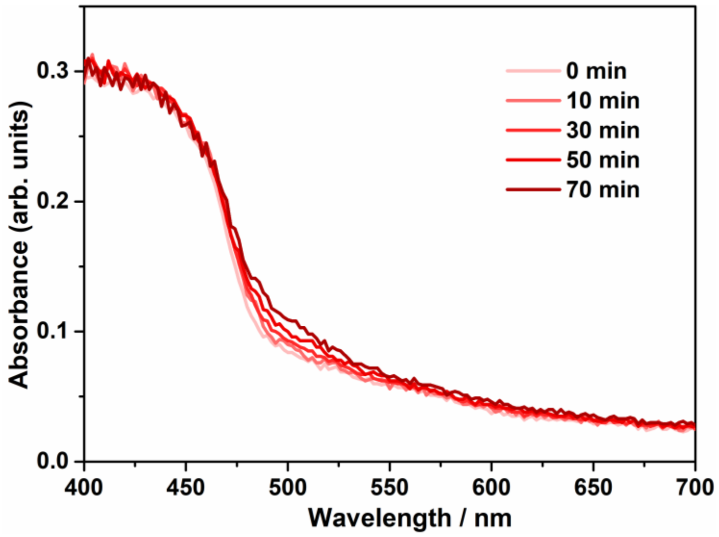

Supplementary Figure 19. Time-dependent UV-Vis spectra of H_2ADC at solid state upon light irradiation.

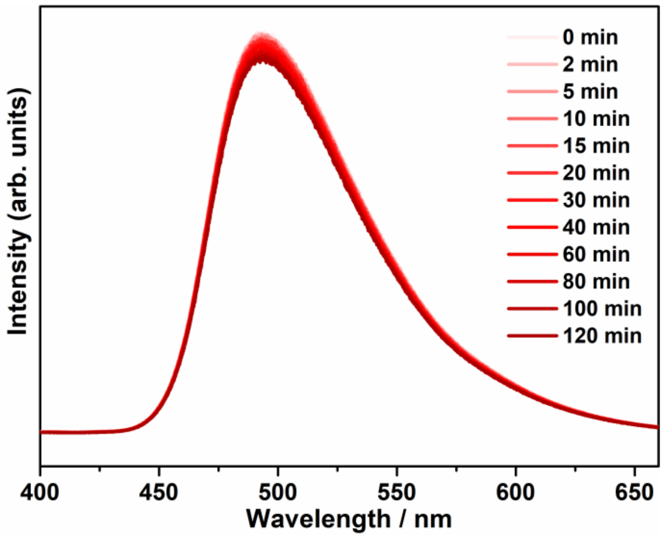

Supplementary Figure 20. Time-dependent photoluminescent spectra of H_2ADC excited at 280 nm.

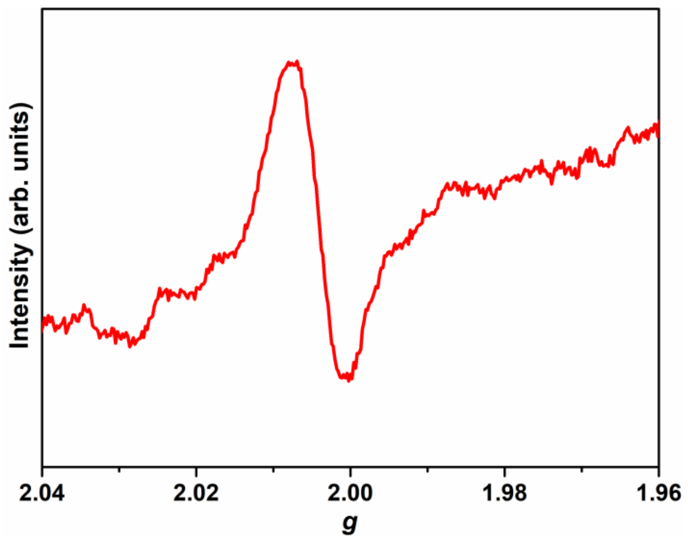

Supplementary Figure 21. ESR spectra of H_2ADC after Xe-lamp light illumination under a frequency of 9.84 GHz.

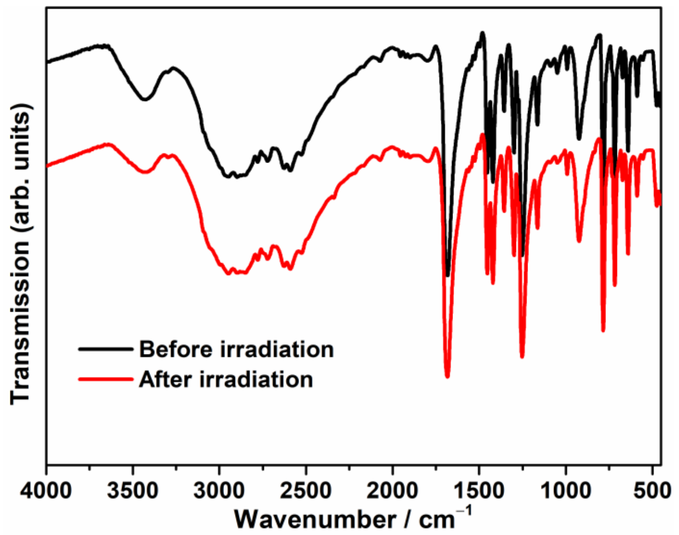

Supplementary Figure 22. IR plots for H_2ADC at 293 K.

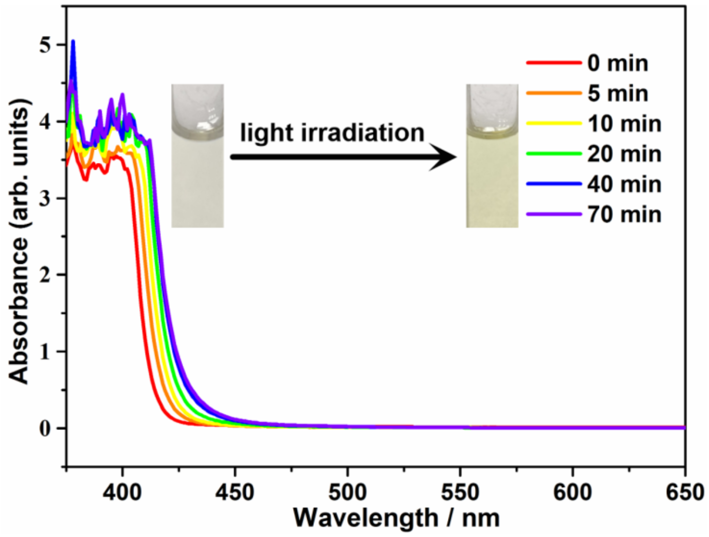

Supplementary Figure 23. Time-dependent UV-Vis spectra of H_2ADC at DMF solvent upon light irradiation.

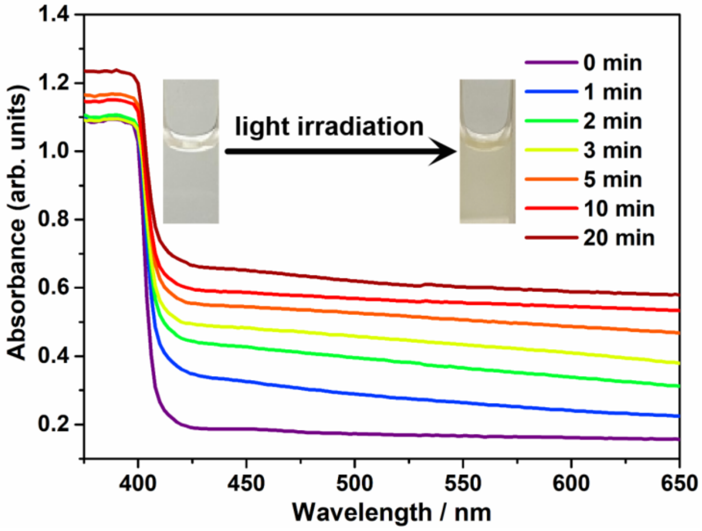

Supplementary Figure 24. Time-dependent UV-Vis spectra of **1** at aqueous solution upon light irradiation.

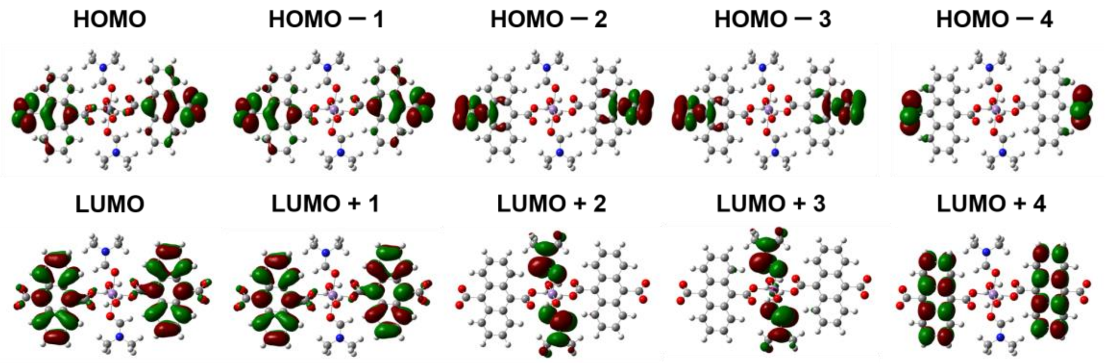

Supplementary Figure 25. HOMO, HOMO−1, HOMO−2, HOMO−3, HOMO−4, LUMO, LUMO+1, LUMO+2, LUMO+3 and LUMO+4 frontier molecular orbitals of compound **1** obtained by DFT calculations.

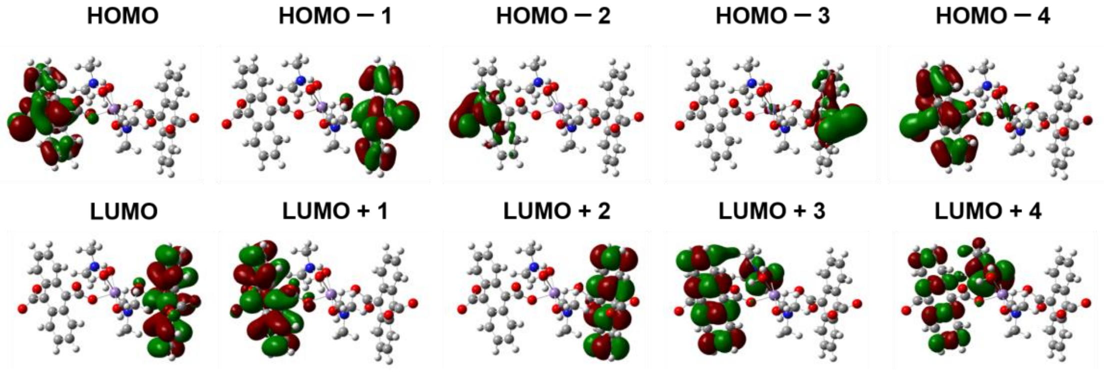

Supplementary Figure 26. HOMO, HOMO−1, HOMO−2, HOMO−3, HOMO−4, LUMO, LUMO+1, LUMO+2, LUMO+3 and LUMO+4 frontier molecular orbitals of compound **1a** obtained by DFT calculations.

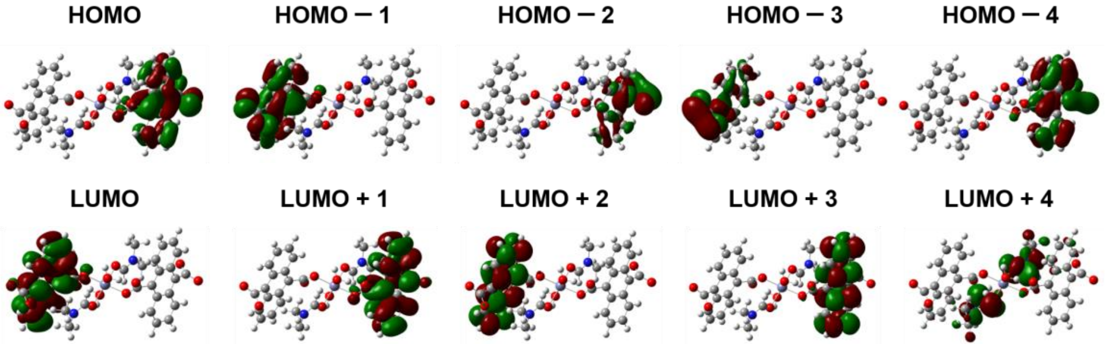

Supplementary Figure 27. HOMO, HOMO−1, HOMO−2, HOMO−3, HOMO−4, LUMO, LUMO+1, LUMO+2, LUMO+3 and LUMO+4 frontier molecular orbitals of compound **2** obtained by DFT calculations.

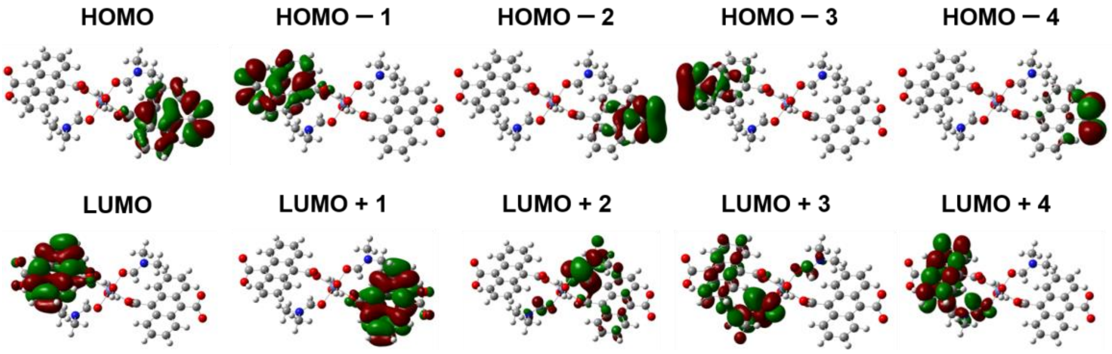

Supplementary Figure 28. HOMO, HOMO−1, HOMO−2, HOMO−3, HOMO−4, LUMO, LUMO+1, LUMO+2, LUMO+3 and LUMO+4 frontier molecular orbitals of compound **3** obtained by DFT calculations.

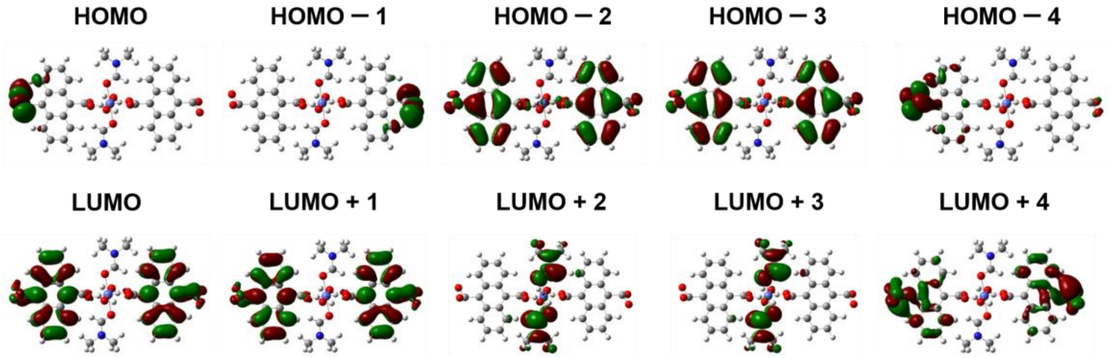

Supplementary Figure 29. HOMO, HOMO−1, HOMO−2, HOMO−3, HOMO−4, LUMO, LUMO+1, LUMO+2, LUMO+3 and LUMO+4 frontier molecular orbitals of compound **4** obtained by DFT calculations.

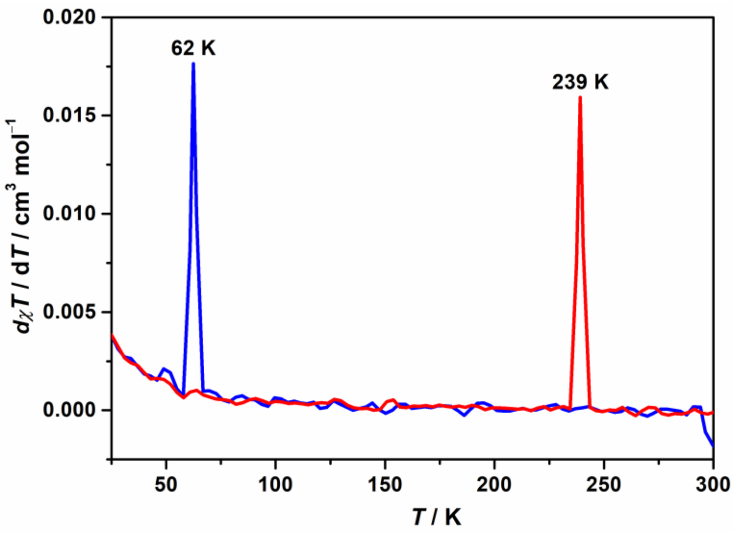

Supplementary Figure 30. The first order derivatives of magnetic susceptibility for **1a** in the cooling (blue) and heating (red) mode.

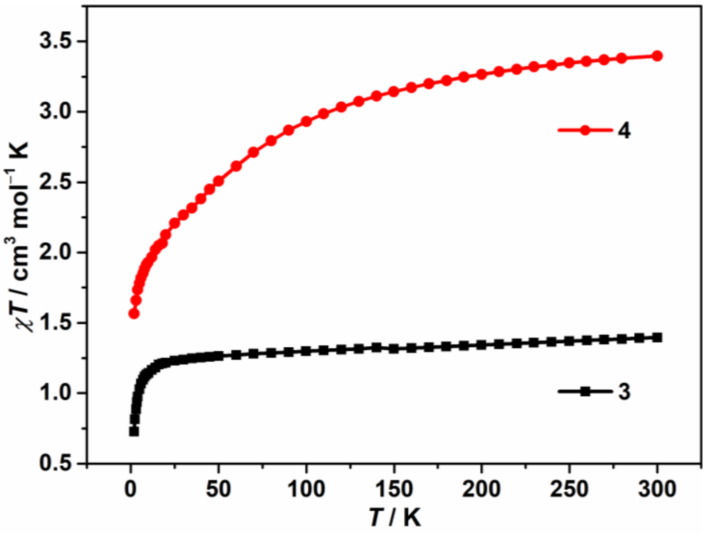

Supplementary Figure 31. Temperature-dependent susceptibilities of compounds **3** and **4** under a direct-current magnetic field of 1000 Oe.

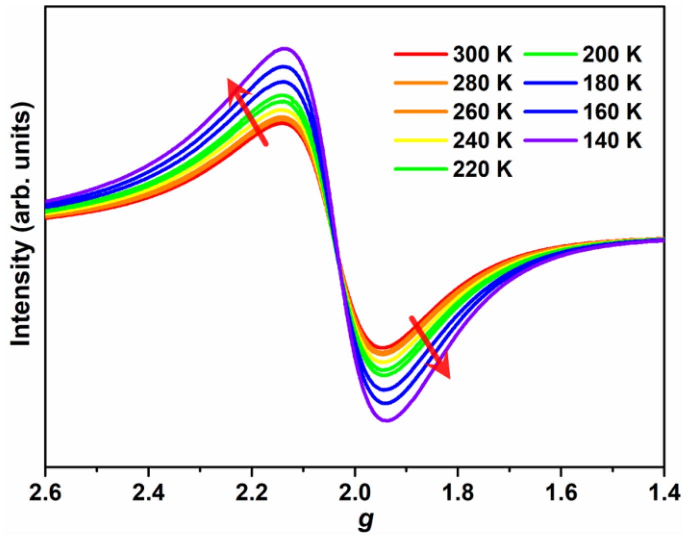

Supplementary Figure 32. ESR spectra of **1** under variable temperatures in the solid state under a frequency of 9.41 GHz.

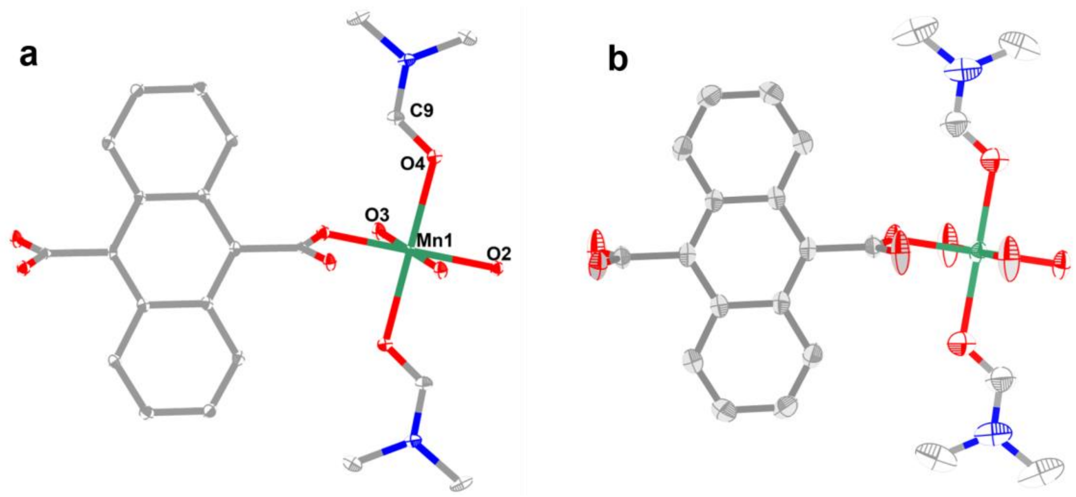

Supplementary Figure 33. Molecular view of **1a** at 50 K (**a**) and 300 K (**b**) with thermal ellipsoids set at 50% probability.

Supplementary Tables

Supplementary Table 1. Crystallographic data for compounds **1**, **1a** and **2**.

	**1**	**1a (300 K)**	**1a (93 K)**	**1a (50 K)**	**2 (293 K)**
Formula	$C_{22}H_{26}N_2MnO_8$	$C_{22}H_{26}N_2MnO_8$	$C_{22}H_{26}N_2MnO_8$	$C_{22}H_{26}N_2MnO_8$	$C_{22}H_{26}N_2ZnO_8$
*M*r (g·mol^{-1})	501.39	501.39	501.39	501.39	511.82
Space group	$P2_1/c$	$P2_1/c$	$P2_1/c$	$P2_1/c$	$P2_1/n$
Crystal system	Monoclinic	Monoclinic	Monoclinic	Monoclinic	Monoclinic
a (Å)	7.4467(5)	7.4277(8)	7.3345(3)	7.3157(5)	7.3224(6)
b (Å)	14.0847(8)	14.0432(15)	14.0017(6)	13.9869(10)	13.9149(10)
c (Å)	11.3810(6)	11.3502(11)	11.2243(5)	11.2142(7)	11.3052(9)
α (°)	90	90	90	90	90
β (°)	107.562(6)	107.531(3)	106.625(10)	106.607(2)	108.641(9)
γ (°)	90	90	90	90	90
V (Å^3)	1138.05(12)	1128.9(2)	1104.34(8)	1099.62(13)	1091.46(16)
Z	2	2	2	2	2
F (000)	522	522	522	522	532
Dc (gcm^{-3})	1.463	1.475	1.508	1.514	1.557
μ (mm^{-1})	0.631	0.636	0.650	0.653	1.178
R_{int}	0.0139	0.0349	0.0393	0.0435	0.0271
	-8≤h≤8	-9≤h≤9	-8≤h≤8	-8≤h≤8	-11≤h≤9
limiting indices	-16≤k≤14	-17≤k≤12	-16≤k≤13	-16≤k≤16	-20≤k≤17
	-13≤l≤13	-13≤l≤13	-13≤l≤12	-12≤l≤13	-14≤l≤17
Collected reflections	5074	12928	11650	12065	8286
Unique reflections	2008	2111	1921	1931	3687
GOF on F^2	1.088	1.115	1.158	1.142	1.003
R_1, wR_2 [$I>2\sigma(I)$]	0.0430 0.1131	0.0479 0.1367	0.0405 0.1216	0.0273 0.0685	0.0420 0.1024
R_1, wR_2 [all data]	0.0500 0.1197	0.0491 0.1373	0.0410 0.1218	0.0311 0.0700	0.0699 0.1203

$^aR_1 = \sum ||F_o| - |F_c|| / \sum |F_o|$. $^b wR_2 = \{\sum[w(F_o^2 - F_c^2)^2] / \sum w(F_o^2)^2\}^{1/2}$.

Supplementary Table 2. Crystallographic data for compounds **3** and **4** at 293 K.

	**3**		**4**	
Formula	$C_{22}H_{26}N_2NiO_8$		$C_{22}H_{26}N_2CoO_8$	
*M*r (g·mol^{-1})	505.16		505.38	
Space group	$P2_1/n$		$P2_1/c$	
Crystal system	Monoclinic		Monoclinic	
a (Å)	7.2927(10)		7.3114(5)	
b (Å)	13.8403(15)		13.8990(10)	
c (Å)	11.2599(15)		11.3171(9)	
α (°)	90		90	
β (°)	108.183(15)		108.717(9)	
γ (°)	90		90	
V (Å^3)	1079.7(3)		1089.24(15)	
Z	2		2	
F (000)	528		526	
Dc (gcm^{-3})	1.554		1.541	
μ (mm^{-1})	0.952		0.841	
R_{int}	0.0589		0.0180	
	-10≤h≤10		-8≤h≤8	
limiting indices	-20≤k≤17		-16≤k≤16	
	-13≤l≤16		-13≤l≤13	
Collected reflections	7596		5809	
Unique reflections	3569		1963	
GOF on F^2	1.002		1.075	
R_1, wR_2 [I>2σ(I)]	0.0574	0.1419	0.0299	0.0744
R_1, wR_2 [all data]	0.0897	0.1656	0.0375	0.0799

$^aR_1 = \sum ||F_o| - |F_c|| / \sum |F_o|$. $^b wR_2 = \{\sum[w(F_o^2 - F_c^2)^2] / \sum w(F_o^2)^2\}^{1/2}$.

Supplementary Table 3. Selected bond lengths (Å) and angles (º) for compound **1** at 293 K.

Compound **1**			
Mn(1)-O(2)	2.1566(14)	C(3)-C(8)	1.516(3)
Mn(1)-O(2)#1	2.1566(14)	C(4)-C(5)	1.429(3)
Mn(1)-O(3)	2.1723(19)	C(5)-C(6)	1.358(3)
Mn(1)-O(3)#1	2.1723(19)	C(6)-C(7)	1.428(4)
Mn(1)-O(4)	2.2162(18)	C(8)-O(1)	1.236(3)
Mn(1)-O(4)#1	2.2162(18)	C(8)-O(2)	1.257(3)
C(1)-C(7)	1.358(3)	C(9)-O(4)	1.247(3)
C(1)-C(2)	1.430(3)	C(9)-N(1)	1.304(3)
C(2)-C(3)	1.407(3)	C(10)-N(1)	1.466(4)
C(2)-C(4)	1.446(3)	C(11)-N(1)	1.464(4)
C(3)-C(4)#2	1.404(2)		
O(2)-Mn(1)-O(2)#1	180	C(4)#2-C(3)-C(2)	121.51(19)
O(2)-Mn(1)-O(3)	91.25(7)	C(4)#2-C(3)-C(8)	118.78(16)
O(2)#1-Mn(1)-O(3)	88.75(7)	C(2)-C(3)-C(8)	119.71(16)
O(2)-Mn(1)-O(3)#1	88.75(7)	C(3)#2-C(4)-C(5)	122.64(17)
O(2)#1-Mn(1)-O(3)#1	91.25(7)	C(3)#2-C(4)-C(2)	119.15(16)
O(3)-Mn(1)-O(3)#1	180	C(5)-C(4)-C(2)	118.20(16)
O(2)-Mn(1)-O(4)	91.66(6)	C(6)-C(5)-C(4)	121.53(19)
O(2)#1-Mn(1)-O(4)	88.34(6)	C(5)-C(6)-C(7)	120.37(19)
O(3)-Mn(1)-O(4)	88.48(7)	C(1)-C(7)-C(6)	120.12(19)
O(3)#1-Mn(1)-O(4)	91.52(7)	O(1)-C(8)-O(2)	125.7(2)
O(2)-Mn(1)-O(4)#1	88.34(6)	O(1)-C(8)-C(3)	117.92(19)
O(2)#1-Mn(1)-O(4)#1	91.66(6)	O(2)-C(8)-C(3)	116.36(17)
O(3)-Mn(1)-O(4)#1	91.52(7)	O(4)-C(9)-N(1)	125.1(3)
O(3)#1-Mn(1)-O(4)#1	88.48(7)	C(9)-N(1)-C(11)	120.7(3)
O(4)-Mn(1)-O(4)#1	180	C(9)-N(1)-C(10)	120.7(3)
C(7)-C(1)-C(2)	121.61(19)	C(11)-N(1)-C(10)	118.6(3)
C(3)-C(2)-C(1)	122.50(17)	C(8)-O(2)-Mn(1)	130.68(13)
C(3)-C(2)-C(4)	119.34(16)	C(9)-O(4)-Mn(1)	122.99(19)
C(1)-C(2)-C(4)	118.16(16)		

Symmetry codes: #1 -x,-y,-z #2 -x+1,-y,-z+1.

Supplementary Table 4. Selected bond lengths (Å) and angles (º) for compound **1a** at 300 K.

Compound **1a** (300 K)			
Mn(1)-O(2)#1	2.1492(16)	C(3)-C(8)	1.514(3)
Mn(1)-O(2)	2.1492(16)	C(4)-C(5)	1.429(3)
Mn(1)-O(3)#1	2.166(2)	C(5)-C(6)	1.354(4)
Mn(1)-O(3)	2.166(2)	C(6)-C(7)	1.429(4)
Mn(1)-O(4)	2.208(2)	C(8)-O(1)	1.241(3)
Mn(1)-O(4)#1	2.208(2)	C(8)-O(2)	1.246(3)
C(1)-C(7)	1.356(4)	C(9)-O(4)	1.257(4)
C(1)-C(2)	1.427(3)	C(9)-N(1)	1.301(4)
C(2)-C(3)	1.403(3)	C(10)-N(1)	1.458(5)
C(2)-C(4)	1.436(3)	C(11)-N(1)	1.458(5)
C(3)-C(4)#2	1.403(3)		
O(2)#1-Mn(1)-O(2)	180	C(4)#2-C(3)-C(2)	121.4(2)
O(2)#1-Mn(1)-O(3)#1	91.27(8)	C(4)#2-C(3)-C(8)	118.77(19)
O(2)-Mn(1)-O(3)#1	88.73(8)	C(2)-C(3)-C(8)	119.83(19)
O(2)#1-Mn(1)-O(3)	88.73(8)	C(3)#2-C(4)-C(5)	122.5(2)
O(2)-Mn(1)-O(3)	91.27(8)	C(3)#2-C(4)-C(2)	119.24(19)
O(3)#1-Mn(1)-O(3)	180	C(5)-C(4)-C(2)	118.24(19)
O(2)#1-Mn(1)-O(4)	88.35(8)	C(6)-C(5)-C(4)	121.5(2)
O(2)-Mn(1)-O(4)	91.65(8)	C(5)-C(6)-C(7)	120.4(2)
O(3)#1-Mn(1)-O(4)	91.72(9)	C(1)-C(7)-C(6)	119.8(2)
O(3)-Mn(1)-O(4)	88.27(9)	O(1)-C(8)-O(2)	125.7(2)
O(2)#1-Mn(1)-O(4)#1	91.65(8)	O(1)-C(8)-C(3)	117.3(2)
O(2)-Mn(1)-O(4)#1	88.35(8)	O(2)-C(8)-C(3)	117.0(2)
O(3)#1-Mn(1)-O(4)#1	88.27(9)	O(4)-C(9)-N(1)	124.8(3)
O(3)-Mn(1)-O(4)#1	91.73(9)	C(9)-N(1)-C(11)	120.2(3)
O(4)-Mn(1)-O(4)#1	180	C(9)-N(1)-C(10)	121.3(3)
C(7)-C(1)-C(2)	121.8(2)	C(11)-N(1)-C(10)	118.5(3)
C(3)-C(2)-C(1)	122.4(2)	C(8)-O(2)-Mn(1)	131.00(16)
C(3)-C(2)-C(4)	119.35(19)	C(9)-O(4)-Mn(1)	122.4(2)
C(1)-C(2)-C(4)	118.24(19)		

Symmetry codes: #1 -x,-y,-z #2 -x+1,-y,-z+1.

Supplementary Table 5. Selected bond lengths (Å) and angles (º) for compound **1a** at 93 K.

Compound **1a** (93K)			
Mn(1)-O(2)#1	2.1505(17)	C(3)-C(8)	1.512(3)
Mn(1)-O(2)	2.1505(17)	C(4)-C(5)	1.434(3)
Mn(1)-O(3)#1	2.1717(17)	C(5)-C(6)	1.356(4)
Mn(1)-O(3)	2.1717(17)	C(6)-C(7)	1.434(4)
Mn(1)-O(4)#1	2.2067(18)	C(8)-O(1)	1.255(3)
Mn(1)-O(4)	2.2067(18)	C(8)-O(2)	1.261(3)
C(1)-C(7)	1.356(4)	C(9)-O(4)	1.251(3)
C(1)-C(2)	1.431(3)	C(9)-N(1)	1.318(4)
C(2)-C(3)	1.400(3)	C(10)-N(1)	1.457(4)
C(2)-C(4)	1.443(4)	C(11)-N(1)	1.455(4)
C(3)-C(4)#2	1.399(3)		
O(2)#1-Mn(1)-O(2)	180	C(4)#2-C(3)-C(2)	121.9(2)
O(2)#1-Mn(1)-O(3)#1	89.82(7)	C(4)#2-C(3)-C(8)	118.5(2)
O(2)-Mn(1)-O(3)#1	90.18(7)	C(2)-C(3)-C(8)	119.6(2)
O(2)#1-Mn(1)-O(3)	90.18(7)	C(3)#2-C(4)-C(5)	122.7(2)
O(2)-Mn(1)-O(3)	89.82(7)	C(3)#2-C(4)-C(2)	119.1(2)
O(3)#1-Mn(1)-O(3)	180	C(5)-C(4)-C(2)	118.2(2)
O(2)#1-Mn(1)-O(4)#1	92.24(7)	C(6)-C(5)-C(4)	121.5(2)
O(2)-Mn(1)-O(4)#1	87.76(7)	C(5)-C(6)-C(7)	120.3(2)
O(3)#1-Mn(1)-O(4)#1	85.59(7)	C(1)-C(7)-C(6)	120.1(2)
O(3)-Mn(1)-O(4)#1	94.41(7)	O(1)-C(8)-O(2)	125.6(2)
O(2)#1-Mn(1)-O(4)	87.76(7)	O(1)-C(8)-C(3)	117.3(2)
O(2)-Mn(1)-O(4)	92.24(7)	O(2)-C(8)-C(3)	117.1(2)
O(3)#1-Mn(1)-O(4)	94.41(7)	O(4)-C(9)-N(1)	124.9(3)
O(3)-Mn(1)-O(4)	85.59(7)	C(9)-N(1)-C(11)	121.4(2)
O(4)#1-Mn(1)-O(4)	180	C(9)-N(1)-C(10)	121.1(3)
C(7)-C(1)-C(2)	121.7(2)	C(11)-N(1)-C(10)	117.5(3)
C(3)-C(2)-C(1)	122.7(2)	C(8)-O(2)-Mn(1)	129.01(16)
C(3)-C(2)-C(4)	119.0(2)	C(9)-O(4)-Mn(1)	120.68(17)
C(1)-C(2)-C(4)	118.2(2)		

Symmetry codes: #1 -x,-y,-z #2 -x+1,-y,-z+1.

Supplementary Table 6. Selected bond lengths (Å) and angles (º) for compound **1a** at 50 K.

Compound **1a** (50K)			
Mn(1)-O(2)#1	2.1522(11)	C(3)-C(8)	1.512(2)
Mn(1)-O(2)	2.1522(11)	C(4)-C(5)	1.434(2)
Mn(1)-O(3)	2.1722(12)	C(5)-C(6)	1.357(2)
Mn(1)-O(3)#1	2.1722(12)	C(6)-C(7)	1.427(2)
Mn(1)-O(4)#1	2.2077(12)	C(8)-O(1)	1.254(2)
Mn(1)-O(4)	2.2077(12)	C(8)-O(2)	1.262(2)
C(1)-C(7)	1.362(2)	C(9)-O(4)	1.249(2)
C(1)-C(2)	1.433(2)	C(9)-N(1)	1.320(2)
C(2)-C(3)	1.402(2)	C(10)-N(1)	1.461(2)
C(2)-C(4)	1.435(2)	C(11)-N(1)	1.460(2)
C(3)-C(4)#2	1.405(2)		
O(2)#1-Mn(1)-O(2)	180	C(2)-C(3)-C(4)#2	121.35(16)
O(2)#1-Mn(1)-O(3)	90.40(5)	C(2)-C(3)-C(8)	119.99(15)
O(2)-Mn(1)-O(3)	89.60(5)	C(4)#2-C(3)-C(8)	118.67(15)
O(2)#1-Mn(1)-O(3)#1	89.60(5)	C(3)#2-C(4)-C(5)	122.25(15)
O(2)-Mn(1)-O(3)#1	90.40(5)	C(3)#2-C(4)-C(2)	119.20(15)
O(3)-Mn(1)-O(3)#1	180	C(5)-C(4)-C(2)	118.53(15)
O(2)#1-Mn(1)-O(4)#1	92.33(4)	C(6)-C(5)-C(4)	121.08(16)
O(2)-Mn(1)-O(4)#1	87.67(4)	C(5)-C(6)-C(7)	120.60(16)
O(3)-Mn(1)-O(4)#1	94.62(5)	C(1)-C(7)-C(6)	120.14(16)
O(3)#1-Mn(1)-O(4)#1	85.38(5)	O(1)-C(8)-O(2)	125.95(16)
O(2)#1-Mn(1)-O(4)	87.67(4)	O(1)-C(8)-C(3)	117.46(15)
O(2)-Mn(1)-O(4)	92.33(4)	O(2)-C(8)-C(3)	116.58(15)
O(3)-Mn(1)-O(4)	85.38(5)	O(4)-C(9)-N(1)	124.85(17)
O(3)#1-Mn(1)-O(4)	94.62(5)	C(9)-N(1)-C(11)	121.59(15)
O(4)#1-Mn(1)-O(4)	180	C(9)-N(1)-C(10)	120.89(15)
C(7)-C(1)-C(2)	121.23(16)	C(11)-N(1)-C(10)	117.51(15)
C(3)-C(2)-C(1)	122.11(16)	C(8)-O(2)-Mn(1)	128.39(11)
C(3)-C(2)-C(4)	119.45(15)	C(9)-O(4)-Mn(1)	120.45(11)
C(1)-C(2)-C(4)	118.43(15)		

Symmetry codes: #1 -x,-y,-z #2 -x+1,-y,-z+1.

Supplementary Table 7. Selected bond lengths (Å) and angles (º) for compound **2** at 293 K.

Compound **2**			
Zn(1)-O(2)#1	2.0572(13)	C(3)-C(8)	1.505(2)
Zn(1)-O(2)	2.0572(13)	C(4)-C(5)	1.429(2)
Zn(1)-O(3)#1	2.0876(16)	C(5)-C(6)	1.353(3)
Zn(1)-O(3)	2.0877(16)	C(6)-C(7)	1.412(3)
Zn(1)-O(4)#1	2.1381(16)	C(8)-O(1)	1.239(2)
Zn(1)-O(4)	2.1382(16)	C(8)-O(2)	1.253(2)
C(1)-C(7)#2	1.350(3)	C(9)-O(4)	1.236(3)
C(1)-C(2)	1.428(2)	C(9)-N(1)	1.294(3)
C(2)-C(3)	1.395(2)	C(10)-N(1)	1.457(3)
C(2)-C(4)#2	1.432(2)	C(11)-N(1)	1.454(3)
C(3)-C(4)	1.399(2)		
O(2)#1-Zn(1)-O(2)	180.000(10)	C(2)-C(3)-C(4)	121.14(15)
O(2)#1-Zn(1)-O(3)#1	88.90(6)	C(2)-C(3)-C(8)	119.81(13)
O(2)-Zn(1)-O(3)#1	91.10(6)	C(4)-C(3)-C(8)	119.05(13)
O(2)#1-Zn(1)-O(3)	91.10(6)	C(3)-C(4)-C(5)	122.08(14)
O(2)-Zn(1)-O(3)	88.90(6)	C(3)-C(4)-C(2)#2	119.32(13)
O(3)#1-Zn(1)-O(3)	180	C(5)-C(4)-C(2)#2	118.60(13)
O(2)#1-Zn(1)-O(4)#1	93.02(6)	C(6)-C(5)-C(4)	120.91(16)
O(2)-Zn(1)-O(4)#1	86.98(6)	C(5)-C(6)-C(7)	120.72(16)
O(3)#1-Zn(1)-O(4)#1	87.93(7)	C(1)#2-C(7)-C(6)	120.24(16)
O(3)-Zn(1)-O(4)#1	92.07(7)	O(1)-C(8)-O(2)	126.23(17)
O(2)#1-Zn(1)-O(4)	86.98(6)	O(1)-C(8)-C(3)	118.09(17)
O(2)-Zn(1)-O(4)	93.02(6)	O(2)-C(8)-C(3)	115.67(16)
O(3)#1-Zn(1)-O(4)	92.07(7)	O(4)-C(9)-N(1)	125.7(3)
O(3)-Zn(1)-O(4)	87.93(7)	C(9)-N(1)-C(11)	121.2(2)
O(4)#1-Zn(1)-O(4)	180	C(9)-N(1)-C(10)	120.9(2)
C(7)#2-C(1)-C(2)	121.66(16)	C(11)-N(1)-C(10)	117.8(3)
C(3)-C(2)-C(1)	122.60(14)	C(8)-O(2)-Zn(1)	130.05(12)
C(3)-C(2)-C(4)#2	119.54(13)	C(9)-O(4)-Zn(1)	121.56(17)
C(1)-C(2)-C(4)#2	117.86(14)		

Symmetry codes: #1 -x+1,-y,-z+1 #2 -x+1,-y,-z+2.

Supplementary Table 8. Selected bond lengths (Å) and angles (º) for compound **3** at 293 K.

Compound **3**			
Ni(1)-O(2)#1	2.0366(17)	C(3)-C(8)	1.504(3)
Ni(1)-O(2)	2.0366(17)	C(4)-C(5)	1.425(3)
Ni(1)-O(3)#1	2.055(2)	C(5)-C(6)	1.355(3)
Ni(1)-O(3)	2.055(2)	C(6)-C(7)	1.410(4)
Ni(1)-O(4)#1	2.0815(19)	C(8)-O(1)	1.240(3)
Ni(1)-O(4)	2.0816(19)	C(8)-O(2)	1.259(3)
C(1)-C(7)	1.356(3)	C(9)-O(4)	1.237(3)
C(1)-C(2)	1.428(3)	C(9)-N(1)	1.301(4)
C(2)-C(3)	1.398(3)	C(10)-N(1)	1.450(4)
C(2)-C(4)	1.429(3)	C(11)-N(1)	1.464(4)
C(3)-C(4)#2	1.401(3)		
O(2)#1-Ni(1)-O(2)	180	C(2)-C(3)-C(4)#2	121.1(2)
O(2)#1-Ni(1)-O(3)#1	91.71(8)	C(2)-C(3)-C(8)	120.16(17)
O(2)-Ni(1)-O(3)#1	88.28(8)	C(4)#2-C(3)-C(8)	118.71(17)
O(2)#1-Ni(1)-O(3)	88.29(8)	C(3)#2-C(4)-C(5)	122.19(19)
O(2)-Ni(1)-O(3)	91.71(8)	C(3)#2-C(4)-C(2)	119.13(17)
O(3)#1-Ni(1)-O(3)	180	C(5)-C(4)-C(2)	118.67(18)
O(2)#1-Ni(1)-O(4)#1	93.30(7)	C(6)-C(5)-C(4)	121.1(2)
O(2)-Ni(1)-O(4)#1	86.70(7)	C(5)-C(6)-C(7)	120.5(2)
O(3)#1-Ni(1)-O(4)#1	92.30(8)	C(1)-C(7)-C(6)	120.4(2)
O(3)-Ni(1)-O(4)#1	87.70(8)	O(1)-C(8)-O(2)	126.5(2)
O(2)#1-Ni(1)-O(4)	86.70(7)	O(1)-C(8)-C(3)	118.0(2)
O(2)-Ni(1)-O(4)	93.30(7)	O(2)-C(8)-C(3)	115.5(2)
O(3)#1-Ni(1)-O(4)	87.69(8)	O(4)-C(9)-N(1)	125.3(3)
O(3)-Ni(1)-O(4)	92.31(8)	C(9)-N(1)-C(10)	121.3(3)
O(4)#1-Ni(1)-O(4)	180	C(9)-N(1)-C(11)	120.9(3)
C(7)-C(1)-C(2)	121.4(2)	C(10)-N(1)-C(11)	117.8(3)
C(3)-C(2)-C(4)	119.75(17)	C(8)-O(2)-Ni(1)	129.63(15)
C(3)-C(2)-C(1)	122.26(19)	C(9)-O(4)-Ni(1)	121.2(2)
C(4)-C(2)-C(1)	117.99(18)		

Symmetry codes: #1 -x+1,-y,-z+1 #2 -x+1,-y,-z.

Supplementary Table 9. Selected bond lengths (Å) and angles (º) for compound **4** at 293 K.

Compound **4**			
Co(1)-O(2)#1	2.0725(14)	C(3)-C(8)	1.502(3)
Co(1)-O(2)	2.0726(14)	C(4)-C(5)	1.426(3)
Co(1)-O(3)#1	2.0773(15)	C(5)-C(6)	1.352(3)
Co(1)-O(3)	2.0773(15)	C(6)-C(7)	1.412(3)
Co(1)-O(4)	2.1199(15)	C(8)-O(1)	1.241(2)
Co(1)-O(4)#1	2.1199(15)	C(8)-O(2)	1.251(2)
C(1)-C(7)	1.351(3)	C(9)-O(4)	1.239(3)
C(1)-C(2)	1.423(3)	C(9)-N(1)	1.306(3)
C(2)-C(3)	1.400(2)	C(10)-N(1)	1.453(3)
C(2)-C(4)	1.428(3)	C(11)-N(1)	1.458(3)
C(3)-C(4)#2	1.399(2)		
O(2)#1-Co(1)-O(2)	180.00(8)	C(4)#2-C(3)-C(2)	121.10(17)
O(2)#1-Co(1)-O(3)#1	90.59(6)	C(4)#2-C(3)-C(8)	118.99(15)
O(2)-Co(1)-O(3)#1	89.41(6)	C(2)-C(3)-C(8)	119.90(15)
O(2)#1-Co(1)-O(3)	89.41(6)	C(3)#2-C(4)-C(5)	122.27(17)
O(2)-Co(1)-O(3)	90.59(6)	C(3)#2-C(4)-C(2)	119.33(15)
O(3)#1-Co(1)-O(3)	180	C(5)-C(4)-C(2)	118.39(16)
O(2)#1-Co(1)-O(4)	93.08(6)	C(6)-C(5)-C(4)	121.22(19)
O(2)-Co(1)-O(4)	86.92(6)	C(5)-C(6)-C(7)	120.50(18)
O(3)#1-Co(1)-O(4)	92.54(7)	C(1)-C(7)-C(6)	120.08(18)
O(3)-Co(1)-O(4)	87.46(7)	O(1)-C(8)-O(2)	126.18(18)
O(2)#1-Co(1)-O(4)#1	86.92(6)	O(1)-C(8)-C(3)	117.86(17)
O(2)-Co(1)-O(4)#1	93.08(6)	O(2)-C(8)-C(3)	115.96(16)
O(3)#1-Co(1)-O(4)#1	87.46(7)	O(4)-C(9)-N(1)	125.2(3)
O(3)-Co(1)-O(4)#1	92.54(7)	C(9)-N(1)-C(10)	121.0(2)
O(4)-Co(1)-O(4)#1	180	C(9)-N(1)-C(11)	120.7(3)
C(7)-C(1)-C(2)	121.77(19)	C(10)-N(1)-C(11)	118.3(2)
C(3)-C(2)-C(1)	122.40(17)	C(8)-O(2)-Co(1)	130.02(12)
C(3)-C(2)-C(4)	119.56(15)	C(9)-O(4)-Co(1)	121.43(17)
C(1)-C(2)-C(4)	118.03(16)		

Symmetry codes: #1 -x,-y+1,-z+1 #2 -x+1,-y+1,-z+2.

Supplementary Table 10. Continuous Shape Measure (CShM) analyses of geometries for compounds **1−4** by SHAPE 2.0 Software.

Geometry	**1**	**1a (300 K)**	**1a (93 K)**	**1a (50 K)**	**2**	**3**	**4**
Hexagon (D_{6h})	32.607	32.541	30.719	30.519	30.951	30.508	30.933
Pentagonal pyramid (C_{5v})	29.948	29.921	29.098	29.012	29.050	28.944	29.191
Octahedron (O_h)	0.047	0.050	0.136	0.149	0.101	0.107	0.095
Trigonal prism (D_{3h})	16.257	16.233	16.090	16.089	16.346	16.363	16.220
Johnson pentagonal pyramid J2 (C_{5v})	33.393	33.371	32.439	32.342	32.310	32.234	32.543

Supplementary Table 11. The H-bond lengths (Å) for **1** at 293 K.

D−H···A	*d* (D−H) (Å)	*d* (H···A) (Å)	*d* (D···A) (Å)	∠(DHA) (deg)
O(3)−H(3A)···O(1)	0.85	1.85	2.677(3)	164
O(3)−H(3B)···O(1)	0.85	2.04	2.754(3)	142

Supplementary Table 12. The H-bond lengths (Å) for **1a** at 300 K.

D−H···A	*d* (D−H) (Å)	*d* (H···A) (Å)	*d* (D···A) (Å)	∠(DHA) (deg)
O(3)−H(3A)···O(1)	0.85	1.92	2.673(3)	146
O(3)−H(3B)···O(1)	0.85	2.18	2.748(3)	125

Supplementary Table 13. The H-bond lengths (Å) for **1a** at 93 K.

D−H···A	*d* (D−H) (Å)	*d* (H···A) (Å)	*d* (D···A) (Å)	∠(DHA) (deg)
O(3)−H(3A)···O(1)	0.85	1.84	2.678(3)	166
O(3)−H(3B)···O(1)	0.85	2.06	2.745(3)	138

Supplementary Table 14. The H-bond lengths (Å) for **1a** at 50 K.

D−H···A	*d* (D−H) (Å)	*d* (H···A) (Å)	*d* (D···A) (Å)	∠(DHA) (deg)
O(3)−H(3A)···O(1)	0.85	1.83	2.679(17)	169
O(3)−H(3B)···O(1)	0.85	2.01	2.745(18)	145

Supplementary Table 15. The H-bond lengths (Å) for **2** at 293 K.

D−H···A	*d* (D−H) (Å)	*d* (H···A) (Å)	*d* (D···A) (Å)	∠(DHA) (deg)
O(3)−H(3A)···O(1)	0.85	1.83	2.644(2)	160
O(3)−H(3B)···O(1)	0.85	2.21	2.814(2)	128

Supplementary Table 16. The complexes with the width of thermal hysteresis loops larger than 100 K.

Molecular formula	$T_{1/2}$↓	$T_{1/2}$↑	ΔT	reproducible	reference
$Rb_{0.73}Mn[Fe(CN)_6]_{0.91}\cdot 1.4H_2O$	147 K	262 K	116 K	√	1
$[FeL_2](BF_4)_2$	350 K	495 K	145 K	√	2
$Rb^{I}_{0.64}Mn^{II}\cdot[Fe^{III}(CN)_6]_{0.88}\cdot 1.7H_2O$	165 K	303 K	138 K	√	3
$[Fe(bpp)_2](CF_3SO_3)_2\cdot H_2O$	147 K	285 K	138 K	√	4
$Rb_{0.94}Mn[Fe(CN)_6]_{0.98}\cdot 2.5H_2O$	185 K	300 K	115 K	√	5
$[FeL_2](BF_4)_2\cdot xH_2O$	360 K	490 K	130 K	×	6
$Co(py_2O)(3,6\text{-}DBQ)_2$	100 K	330 K	230 K	×	7
$[Fe(qsal)_2]NCSe\cdot MeOH$	212 K	352 K	140 K	×	8
$[Fe(qsal)_2]NCSe\cdot CH_2Cl_2$	212 K	392 K	180 K	×	
$[Fe(hyetrz)_3](anion)_2\cdot 3H_2O$	100 K	370 K	270 K	×	9
$[Fe(qsal\text{-}I)_2]OTf\cdot EtOH$	139 K	252 K	113 K	×	10
$[Co(C12\text{-}terpy)_2](BF_4)_2$	258 K	≧400 K	≧142 K	×	11
$[Fe(qsal)_2]NCSe\cdot DMSO$	209 K	324 K	115 K	×	12
$[Fe(qnal\text{-}OMe)_2]BPh_4\cdot 2MeOH$	194 K	304 K	110 K	×	13
$[Fe(1\text{-}BPP\text{-}COOC_2H_5)_2](ClO_4)_2\cdot CH_3CN$	183 K	284 K	101 K	×	14

Supplementary References

1 Ohkoshi, S. i., Matsuda, T., Tokoro, H. & Hashimoto, K. A Surprisingly Large Thermal Hysteresis Loop in a Reversible Phase Transition of $Rb_xMn[Fe(CN)_6]_{(x+2)/3}\times zH_2O$. *Chem. Mater.* **17**, 81−84 (2005).

2 Bushuev, M. B. et al. A mononuclear iron(II) complex: cooperativity, kinetics and activation energy of the solvent-dependent spin transition. *Dalton Trans.* **45**, 107−120 (2016).

3 Tokoro, H., Miyashita, S., Hashimoto, K. & Ohkoshi, S.-i. Huge thermal hysteresis loop and a hidden stable phase in a charge-transfer phase transition of $Rb_{0.64}Mn[Fe(CN)_6]_{0.88}\cdot 1.7H_2O$. *Phys. Rev. B* **73**, 172415 (2006).

4 Buchen, T., Gütlich, P., Sugiyarto, K. H. & Goodwin, H. A. High-Spin→Low-Spin Relaxation in $[Fe(bpp)_2](CF_3SO_3)_2\cdot H_2O$ after LIESST and Thermal Spin-State Trapping-Dynamics of Spin Transition Versus Dynamics of Phase Transition. *Chem. Eur. J.* **2**, 1134−1138 (1996).

5 Azzolina, G. et al. Single laser shot photoinduced phase transition of rubidium manganese hexacyanoferrate investigated by X-ray diffraction. *Eur. J. Inorg. Chem.* **2019**, 3142−3147 (2019).

6 Bushuev, M. B. et al. Unprecedented bistability domain and interplay between spin crossover

and polymorphism in a mononuclear iron(II) complex. *Dalton Trans.* **43**, 3906−3910 (2014).

7 Jung, O. S., Jo, D. H., Lee, Y. A., Conklin, B. J. & Pierpont, C. G. Bistability and Molecular Switching for Semiquinone and Catechol Complexes of Cobalt. Studies on Redox Isomerism for the Bis(pyridine) Ether Series $Co(py_2X)(3,6\text{-}DBQ)_2$, X = O, S, Se, and Te. *Inorg. Chem.* **36**, 19−24 (1997).

8 Hayami, S., Gu, Z. Z., Yoshiki, H., Fujishima, A. & Sato, O. Iron(III) Spin-Crossover Compounds with a Wide Apparent Thermal Hysteresis around Room Temperature. *J. Am. Chem. Soc.* **123**, 11644−11650 (2001).

9 Garcia, Y. et al. Non-classical Fe^{II} spin-crossover behaviour leading to an unprecedented extremely large apparent thermal hysteresis of 270 K: application for displays. *J. Mater. Chem.* **7**, 857−858 (1997).

10 Phonsri, W. et al. Solvent modified spin crossover in an iron(III) complex: phase changes and an exceptionally wide hysteresis. *Chem. Sci.* **8**, 3949−3959 (2017).

11 Han, Y. & Huynh, H. V. Pyrazolin-4-ylidenes: a new class of intriguing ligands. *Dalton Trans.* **40**, 2141−2147 (2011).

12 Hayami, S. et al. Iron(III) spin transition compound with a large thermal hysteresis. *J. Radioanal. Nucl. Chem.* **255**, 443−447 (2003).

13 Nakaya, M. et al. Spin-crossover and LIESST Effect for Iron(III) Complex Based on π–π Stacking by Coordination Programming. *Chem. Lett.* **43**, 1058−1060 (2014).

14 Senthil Kumar, K. et al. Bi-stable spin-crossover characteristics of a highly distorted $[Fe(1\text{-}BPP\text{-}COOC_2H_5)_2](ClO_4)_2\cdot CH_3CN$ complex. *Dalton Trans.* **48**, 3825−3830 (2019).
